# Prenatal Nitrate Intake from Drinking Water and Selected Birth Defects in Offspring of Participants in the National Birth Defects Prevention Study

**DOI:** 10.1289/ehp.1206249

**Published:** 2013-06-14

**Authors:** Jean D. Brender, Peter J. Weyer, Paul A. Romitti, Binayak P. Mohanty, Mayura U. Shinde, Ann M. Vuong, Joseph R. Sharkey, Dipankar Dwivedi, Scott A. Horel, Jiji Kantamneni, John C. Huber, Qi Zheng, Martha M. Werler, Katherine E. Kelley, John S. Griesenbeck, F. Benjamin Zhan, Peter H. Langlois, Lucina Suarez, Mark A. Canfield, the National Birth Defects Prevention Study

**Affiliations:** 1 Department of Epidemiology and Biostatistics, School of Rural Public Health, Texas A&M Health Science Center, College Station, Texas, USA; 2 Center for Health Effects of Environmental Contamination, and; 3 Department of Epidemiology, College of Public Health, University of Iowa, Iowa City, Iowa, USA; 4 Department of Biological and Agricultural Engineering, Texas A&M University, College Station, Texas, USA; 5 Department of Health Promotion and Community Health Sciences, School of Rural Public Health, Texas A&M Health Science Center, College Station, Texas, USA; 6 Slone Epidemiology Center, Boston University, Boston, Massachusetts, USA; 7 III Marine Expeditionary Force, Okinawa, Japan; 8 Department of Geography, Texas State University, San Marcos, Texas, USA; 9 Texas Department of State Health Services, Austin, Texas, USA

## Abstract

Background: Previous studies of prenatal exposure to drinking-water nitrate and birth defects in offspring have not accounted for water consumption patterns or potential interaction with nitrosatable drugs.

Objectives: We examined the relation between prenatal exposure to drinking-water nitrate and selected birth defects, accounting for maternal water consumption patterns and nitrosatable drug exposure.

Methods: With data from the National Birth Defects Prevention Study, we linked addresses of 3,300 case mothers and 1,121 control mothers from the Iowa and Texas sites to public water supplies and respective nitrate measurements. We assigned nitrate levels for bottled water from collection of representative samples and standard laboratory testing. Daily nitrate consumption was estimated from self-reported water consumption at home and work.

Results: With the lowest tertile of nitrate intake around conception as the referent group, mothers of babies with spina bifida were 2.0 times more likely (95% CI: 1.3, 3.2) to ingest ≥ 5 mg nitrate daily from drinking water (vs. < 0.91 mg) than control mothers. During 1 month preconception through the first trimester, mothers of limb deficiency, cleft palate, and cleft lip cases were, respectively, 1.8 (95% CI: 1.1, 3.1), 1.9 (95% CI: 1.2, 3.1), and 1.8 (95% CI: 1.1, 3.1) times more likely than control mothers to ingest ≥ 5.42 mg of nitrate daily (vs. < 1.0 mg). Higher water nitrate intake did not increase associations between prenatal nitrosatable drug use and birth defects.

Conclusions: Higher water nitrate intake was associated with several birth defects in offspring, but did not strengthen associations between nitrosatable drugs and birth defects.

Citation: Brender JD, Weyer PJ, Romitti PA, Mohanty BP, Shinde MU, Vuong AM, Sharkey JR, Dwivedi D, Horel SA, Kantamneni J, Huber JC Jr., Zheng Q, Werler MM, Kelley KE, Griesenbeck JS, Zhan FB, Langlois PH, Suarez L, Canfield MA, and the National Birth Defects Prevention Study. 2013. Prenatal nitrate intake from drinking water and selected birth defects in offspring of participants in the National Birth Defects Prevention Study. Environ Health Perspect 121:1083–1089; http://dx.doi.org/10.1289/ehp.1206249

## Introduction

Nitrate is one of the most widespread chemical contaminants in aquifers around the world (Spalding and Exner 1993). Results from several epidemiologic studies have suggested an association between prenatal exposure to nitrates in drinking water and birth defects in offspring, including neural tube defects (NTDs) (Brender et al. 2004; Croen et al. 2001; Dorsch et al. 1984), central nervous system defects overall (Arbuckle et al. 1988), oral cleft defects (Dorsch et al. 1984), musculoskeletal defects (Dorsch et al. 1984), and congenital heart defects (Cedergren et al. 2002). In these studies, exposure was assigned on the basis of nitrate levels detected in drinking-water sources without further estimating individual consumption of nitrate from such sources. It is noteworthy that previous associations observed between birth defects and nitrates in drinking water were often observed at levels below the current allowable maximum contaminant level for nitrate (10 mg/L as nitrate-nitrogen or 45 mg/L as total nitrate) set by the U.S. Environmental Protection Agency (National Primary Drinking Water Regulations 2010).

Once ingested and absorbed, approximately 25% of nitrate is secreted in saliva (Mensinga et al. 2003), where about 20% is converted to nitrite by bacteria in the mouth (Spiegelhalder et al. 1976). This endogenously formed nitrite, along with nitrite from dietary and drinking-water sources, can react with nitrosatable compounds such as amine- and amide-containing drugs to form *N-*nitroso compounds in the stomach (Gillatt et al. 1985). *N-*Nitroso compounds have been found to be teratogens in animal models (Nagao et al. 1991; Platzek et al. 1983). These compounds are formed to a greater extent in the presence of a nitrosatable compound if nitrite concentration is high (Choi 1985); and when combined with higher nitrite, nitrosatable compounds have been reported to be more strongly associated with exencephaly and skeletal malformations in mice (Teramoto et al. 1980) and with NTDs (Brender et al. 2004, 2011b) and other types of birth defects in humans (Brender et al. 2012). In a small case–control study of Mexican-American women, nitrosatable drug exposure was more strongly associated with NTDs in offspring of women whose drinking-water nitrate measured ≥ 3.5 mg/L than among births to women with lower measured nitrate in their drinking water (Brender et al. 2004).

The objectives of our study were to *a*) examine the relation between prenatal exposure to drinking-water nitrate and birth defects in offspring (selected from defect groups previously associated with higher nitrate in drinking water), accounting for maternal water consumption patterns; and *b*) investigate whether higher daily exposure to drinking-water nitrate or total nitrite that included contributions from diet and drinking water strengthened associations between prenatal exposure to nitrosatable drugs and selected birth defects in offspring.

## Methods

*Study population and design.* To address the study objectives, we used data from the Iowa and Texas sites of the National Birth Defects Prevention Study (NBDPS), an ongoing population-based case–control study of birth defects in the United States (includes sites in 10 states) that began in 1997 (Yoon et al. 2001). The Iowa and Texas sites identify deliveries with major birth defects from live births, stillbirths, and elective terminations as part of their population-based birth defect surveillance. In the NBDPS, case classification is standardized, and clinical information on potentially eligible births is evaluated by a clinical geneticist at each study site and also independently reviewed by one or more other clinical geneticists. For the present study, women with estimated dates of delivery from 1 October 1997 through 31 December 2005 who had deliveries with an NTD, oral cleft, limb deficiency, or congenital heart defect were included. Control infants (live births without any major congenital malformations and whose mothers resided in the study area at delivery) were randomly selected from live birth certificates in Iowa and from hospital delivery records in Texas (proportional to the number of births in each hospital in the geographic regions of study). These comparison infants served as controls for all case groups. The institutional review boards (IRBs) at each NBDPS site and the Centers for Disease Control and Prevention approved the NBDPS study protocol, and the IRBs at the University of Iowa, Texas A&M University, and Texas Department of State Health Services also approved the present project.

*Data collection.* After providing informed consent, case and control mothers were interviewed in English or Spanish by female interviewers using a computer-assisted telephone interview (Yoon et al. 2001). Mothers were questioned about their use of prescription and over-the-counter medications during the index pregnancy, vitamin supplements taken, diet, beverage consumption, work characteristics, and water use. Residential histories were collected for the period 3 months before conception through pregnancy, including the month/year that the mother started and stopped living in each location. A water module was added to the NBDPS interview in 1999, and questions about personal water use were asked of all mothers beginning in 2000, including sources (private well, unfiltered tap, filtered tap, bottled, other); presence and type of filtration; quantity of water drank at home and at work or school on an average day; and any changes including month/year of change in source or quantity of drinking water consumed. Only women who completed the water module were included in the water nitrate analyses, and their estimated dates of delivery ranged from 1998 through 2005.

*Assessment of nitrate in municipal tap water.* After maternal residential addresses were geocoded, we used an approach developed by the Water Subcommittee of the NBDPS Environmental/Occupational Work Group to link geocoded addresses to municipal water supplies. This included *a*) linking geocoded maternal addresses to public water utilities that had digitized boundary maps available; *b*) if utility boundary maps were not available, linking maternal addresses to water utilities using census place names (census place city boundaries were identified through linkage of municipal water system names to census place names); and *c*) contacting water utilities to confirm whether they provided water for maternal addresses that could not be matched using the first two approaches.

Under the federal Safe Drinking Water Act (SDWA 1974), public water supplies using groundwater are required to sample annually for nitrate, and surface water utilities are initially required to sample quarterly, then annually. In Iowa, SDWA and other public water supply data are maintained by the Center for Health Effects of Environmental Contamination at the University of Iowa (Iowa City, IA, USA). In Texas, routine monitoring data for drinking-water nitrate were obtained from the Texas Commission on Environmental Quality (Austin, TX, USA); public water suppliers are required by Texas law to report water monitoring results to this state agency.

Water samples taken during the actual dates of residence during 1 month before conception (B1) through the end of the third month of pregnancy (P3) were given the highest priority for inclusion and averaged if more than one sample result was available. If sample results for this period (B1P3) were unavailable, results were selected, in order of priority, as *a*) any results of samples up to 12 months before the start of B1 through 12 months after the end of P3, or *b*) results of samples taken closest to the earliest date of B1 and results closest to the last day of P3. Using the same approach, we also obtained water nitrate estimates for 1 month before through 1 month postconception (B1P1) for analyses involving NTDs to better reflect the critical exposure window for these defects.

*Assessment of nitrate in bottled water.* Analyses of maternal responses to water use indicated that 341 Iowa and 1,069 Texas mothers (with deliveries having the specified birth defects in this project or control births) reported using bottled water exclusively near the beginning of pregnancy, and a large number of participants in both states reported drinking bottled water in addition to tap water. To estimate exposure to nitrate in bottled water, we conducted a bottled water survey in Iowa and Texas from January through May 2010 in which representative samples of bottled water were collected in major metropolitan and municipal areas that women resided in or nearby. In addition, dispensed waters sold by the gallon were obtained in Iowa stores and in Texas stores, water mills, and kiosks. All samples were tested for nitrate at the State Hygienic Laboratory at The University of Iowa with U.S. Environmental Protection Agency Method 300.0 (Pfaff 1993). Median values were assigned for each city based on multiple bottled water samples collected and respective test results. These median levels were assigned to residents of that city; for cities where bottled water was not collected, the median level of the closest city where water was collected was used.

*Estimation of nitrate in private well water.* Residential addresses of Texas mothers reporting drinking water from private wells were linked to the relevant aquifers. Nearly one-half of the reported private wells were located in the Ogallala Aquifer, with the other reported wells mainly located in five additional major Texas aquifers, including the Edwards-Trinity, Trinity, Carrizo-Wilcox, Gulf Coast, and Hueco-Mesilla Bolson aquifers. We modeled groundwater flow and nitrate transport in these major aquifers and estimated the temporal dynamics of nitrate level at private well locations during the index pregnancies. The modeling effort for individual wells (based on the hydrogeology and the spatial scale of the aquifers) was done separately using two different models: *a*) MODFLOW-MT3DMS (McDonald and Harbaugh 1988; Zheng and Wang 1988) and *b*) HYDRUS-PHRREQC (HP1) model (Jacques and Šim°unek 2005). The wells in the Ogallala Aquifer were modeled using the MODFLOW-MT3DMS because this aquifer encompassed nearly one-half of the private well users, and spanned a large area, which required large scale modeling. Wells in other aquifers were modeled using the HP1 model because the private well users in these aquifers were either localized (e.g., Hueco-Mesilla Bolson, Trinity) or located on a scattered aquifer such as the Seymour Aquifer. The Seymour Aquifer is known as a scattered aquifer because it is in separate areas of erosional remnants of the Seymour Formation of Pleistocene age in parts of 20 Texas counties. Each model was run for 4–9 years depending on the case or control dates of B1P3 and was validated using available historical sampling data from wells in the respective areas. Daily nitrate concentrations obtained from the models were averaged for the respective exposure windows of each Texas mother who reported drinking private well water.

*Estimation of daily intake of nitrate from drinking water.* Nitrate levels in drinking water varied considerably by source. Median levels for bottled water, public water supplies, and private wells (estimated through modeling) were respectively 0.33, 5.0, and 17.6 mg/L as nitrate. For mothers living in more than one residence during the two exposure windows of interest, average nitrate levels from reported drinking-water sources at each residence were obtained and weighted by number of months lived at each address. We developed a program for estimating daily intake of nitrate from drinking water during the exposure windows, using STATA® (Release 11; StataCorp, College Station, TX) that took into account the reported sources of drinking water with respective nitrate concentrations and quantity consumed at home and work, use of water filters and type, consumption of tea and coffee, and any reported changes in water consumption or source during 1 month preconception through the first trimester. We developed two environmental exposure metrics including daily intake of nitrate from drinking water (milligrams) during B1P1 that was used in all analyses of NTDs, and water nitrate intake during B1P3 for analyses of heart, limb, and oral cleft defects. Nitrate intake from drinking-water sources was categorized into tertiles for each exposure period based on the control mothers’ distributions. We were able to estimate daily intake of nitrate from these sources for 87% of case mothers and 88% of control mothers who completed the water module of the NBDPS interview. Reasons for nonlinkage included nitrate in drinking water of private well users not estimated (9% of the Iowa cases/controls) and insufficient/missing addresses or an address outside the United States during the exposure windows of interest.

*Classification of nitrosatable drugs.* In the NBDPS interview, mothers were questioned about prescription and nonprescription drugs used (including start and stop dates) for specific illnesses and disorders and were also prompted for specific products. Methods used to classify drugs with respect to nitrosatability have been described in detail in previous publications (Brender et al. 2011a, 2011b). Briefly, the active ingredients of reported medications used were identified, cross-referenced against previously compiled lists of nitrosatable medicinal compounds (Brambilla and Martelli 2007; McKean-Cowdin et al. 2003), and categorized based on the presence of amine (secondary or tertiary) and amide functional groups in their molecular structures. We focused on exposure to any nitrosatable drugs during the month before and after conception in relation to NTDs and during the first trimester for the other birth defects. Approximately 24% of the control mothers in the NBDPS took one or more nitrosatable drugs during the first trimester (Brender et al. 2011a). The most commonly taken nitrosatable drugs included certain types of antiemetic medications, decongestants, antihistamines, and anti-infectives that contained secondary amines, tertiary amines, or amides as part of their molecular structures.

*Estimation of total nitrite exposure.* To estimate daily intake of nitrate and nitrite from dietary sources, we used a combination of sources, including *a*) the 58-item food frequency questionnaire (FFQ) that elicited information about dietary intake during the year before pregnancy and that was adapted from the short Willett FFQ (Willett et al. 1985), and *b*) additional detailed questions about consumption of breakfast cereals from 3 months before to the end of pregnancy. Procedures were described in detail in a previous publication (Griesenbeck et al. 2009b); briefly, *a*) weighted means for nitrates and nitrites (milligrams/100 g) were calculated for each food item based on the relevant literature; *b*) the respective means were multiplied by the serving size (grams) assigned to each food; *c*) nitrates and nitrites in each serving size were multiplied by the number of servings by month; and *d*) nitrates and nitrites across all food items were summed and then divided by 30 to obtain daily intake of dietary nitrate and nitrite (milligrams). Using the formula suggested by Choi (1985), we estimated total nitrite exposure from food and water as the sum of dietary nitrite intake and 5% of estimated nitrate intake from diet and water sources. Total nitrite intake was further categorized into tertiles based on the control mothers’ distributions. In this population, median contributions of food and drinking-water nitrate to daily intake of nitrate were 94% and 6%, respectively. Approximately 97% and 3% of total nitrite exposure was from food and drinking water, respectively.

*Statistical analysis.* To account for correlation of nitrate intake by geographic location, mixed-effects (random-effects) models for logistic regression were used with mothers nested within cities of residence (nearest city, if rural address) (Goldstein 2010). Mothers in the lowest tertile of nitrate intake from drinking water during B1P1 for analyses of NTDs and B1P3 for the other birth defects served as the referent categories. For limb deficiencies, oral cleft defects, and congenital heart defects, we restricted analyses to isolated birth defects. Covariables were selected *a priori* and based on the literature, and only those cases and controls for which complete data on all pertinent covariables in each analysis were included. For NTDs, covariables included maternal race/ethnicity, education, study site, and any folic acid supplementation during B1P1. In addition to maternal race/ethnicity, education, and study site, covariables for analyses of oral clefts also included maternal age, any smoking 1 month before conception through the first trimester, and folic acid supplementation during the first trimester. Covariables for analyses of limb deficiencies included maternal race/ethnicity, education, age, study site, and multivitamin supplementation during the first trimester. For heart defects, maternal race/ethnicity, education, smoking, study site, and multivitamin supplementation during the first trimester were incorporated into the logistic models. The associations between tertile of prenatal nitrate intake from drinking water and birth defects in offspring were assessed for linear trend by treating the three levels of nitrate intake as a continuous variable in the logistic model and testing the significance of linearity with the *z*-test in STATA® (equivalent to the Wald chi-square test).

As part of a sensitivity analysis, we repeated the above analyses for the subset of participants who reported drinking only municipal tap water during the period around conception and the first trimester. We also examined the association between measured nitrate (milligrams per liter) in municipal water and selected birth defects for which we used the cut points reported by Croen et al. (2001) and Dorsch et al. (1984) (< 5 mg/L, 5–15 mg/L, and > 15 mg/L).

Nitrosatable drug exposure (any vs. none) during B1P1 and the first trimester was stratified by tertiles of nitrate intake from drinking water and by total nitrite from food and water sources. In analyses involving total nitrite, we excluded women with daily caloric intakes of < 500 or > 5,000 kcal, and also adjusted the odds ratios (ORs) for total energy intake (kilocalories per day). We tested for departure from additivity (biologic interaction) in these associations using a statistical program developed by Andersson et al. (2005) that was adapted for STATA®. This program calculated the relative excess risk due to interaction (RERI) and attributable proportion due to interaction (AP) (and their respective 95% CIs). Departures from additive effects were considered present if the confidence intervals of either measure excluded zero. To assess multiplicative interaction, the product terms of any nitrosatable drug use with water nitrate and total nitrite intake were included in the logistic models, and multiplicative interaction was considered present if the *p*-value associated with the interaction term was < 0.05.

## Results

Maternal interviews for offspring with estimated dates of delivery from 1997 through 2005 numbered 317 with NTDs, 177 with limb deficiencies, 654 with oral cleft defects, 2,011 with congenital heart defects, and 1,551 unaffected live births. Maternal participation rates for births with NTDs, limb deficiencies, oral clefts, congenital heart defects, and controls were, respectively, 66%, 72%, 74%, 62%, and 64%. Median time from estimated date of delivery to maternal interview ranged from 9 months for control mothers to 13 months for women with NTD-affected pregnancies. Table 1 shows the characteristics of the case and control mothers. Among participants who completed the water module questions, the proportions of control mothers and mothers of babies with heart defects were similar with respect to usual home sources of drinking water. In contrast, mothers of babies with NTDs, limb deficiencies, and oral clefts were more likely than control mothers to report drinking municipal tap water.

**Table 1:** Selected characteristics of Iowa and Texas case mothers and control mothers in the National Birth Defects Prevention Study, 1997–2005 [*n* (%)].

Characteristic	Controls (*n*=1,551)	Cases			
		NTDs (*n*=317)	Limb deficiencies (*n*=177)	Oral cleft defects (*n*=654)	Heart defects (*n*=2,011)
Race/ethnicity
Non-Hispanic white	901 (58.2)	165 (52.2)	93 (52.5)	393 (60.2)	1,033 (51.5)
Non-Hispanic black	27 (1.7)	9 (2.9)	5 (2.8)	12 (1.8)	60 (3.0)
Hispanic	555 (35.9)	132 (41.8)	67 (37.9)	218 (33.4)	833 (41.5)
Asian/Pacific Islander	21 (1.4)	1 (0.3)	2 (1.1)	12 (1.8)	19 (0.9)
All others	44 (2.8)	9 (2.8)	10 (5.7)	18 (2.8)	62 (3.1)
Missing	3	1	0	1	4
Education (years)
<12	286 (18.8)	64 (20.3)	27 (15.6)	138 (21.3)	408 (20.6)
12	443 (29.2)	87 (27.6)	57 (33.0)	192 (29.7)	574 (29.0)
13–15	436 (28.7)	105 (33.3)	57 (32.9)	186 (28.7)	606 (30.6)
> 15	353 (23.3)	59 (18.7)	32 (18.5)	131 (20.2)	390 (19.7)
Missing	33	2	4	7	33
Age at delivery (years)
<18	95 (6.1)	11 (3.5)	7 (4.0)	29 (4.4)	98 (4.9)
18–19	130 (8.4)	29 (9.1)	19 (10.7)	61 (9.3)	159 (7.9)
20–24	380 (24.5)	79 (24.9)	48 (27.1)	208 (31.8)	535 (26.6)
25–29	453 (29.2)	100 (31.5)	55 (31.1)	170 (26.0)	551 (27.4)
30–34	344 (22.2)	68 (21.5)	35 (19.8)	114 (17.4)	446 (22.2)
> 34	149 (9.6)	30 (9.5)	13 (7.3)	72 (11.0)	222 (11.0)
Study center
Iowa	759 (48.9)	146 (46.1)	80 (45.2)	306 (46.8)	769 (38.2)
Texas	792 (51.1)	171 (53.9)	97 (54.8)	348 (53.2)	1,242 (61.8)
Smoking^*a*^
No	1,199 (78.7)	259 (82.2)	132 (76.3)	471 (72.6)	1,548 (78.1)
Yes	324 (21.3)	56 (17.8)	41 (23.7)	178 (27.4)	433 (21.9)
Missing/out of range	28	2	4	5	30
Nitrosatable drug exposure^*b*^
No	1,166 (77.6)	216 (70.8)	120 (71.9)	482 (76.4)	1,475 (76.2)
Yes	336 (22.4)	89 (29.2)	47 (28.1)	149 (23.6)	460 (23.8)
Total daily nitrite intake^*c*^
≤ 4.78mg/day	726 (66.1)	145 (62.5)	72 (55.8)	334 (68.2)	1,004 (63.5)
>4.78mg/day	372 (33.9)	87 (37.5)	57 (44.2)	156 (31.8)	578 (36.5)
Multivitamin use^*d*^
No	206 (13.6)	33 (10.6)	22 (12.9)	100 (15.7)	304 (15.5)
Yes	1,308 (86.4)	277 (89.4)	148 (87.1)	537 (84.3)	1,658 (84.5)
Missing	37	7	7	17	49
Usual home source of drinking water^*e*^
Tap water, municipal	738 (58.3)	173 (64.3)	96 (64.0)	354 (61.7)	1,011 (56.3)
Tap water, private well	72 (5.7)	19 (7.1)	14 (9.3)	42 (7.3)	99 (5.5)
Bottled water exclusively	455 (36.0)	77 (28.6)	40 (26.7)	178 (31.0)	685 (38.2)
Not available^*f*^	286	48	27	80	216
^***a***^Any smoking between date of conception and end of first trimester. ^***b***^Exposure during the first trimester of pregnancy. ^***c***^Total daily nitrite intake=5% (drinking water nitrate+dietary nitrate)+dietary nitrite. ^***d***^Use during the first trimester of pregnancy. ^***e***^Reported primary drinking water source at the beginning of pregnancy. ^***f***^Water module questions were added in 1999.					

Numbers of births with complete information for maternal daily nitrate intake from water sources and other covariables were 227, 94, 415, 1,046, and 1,105, respectively, for all NTDs, isolated limb deficiencies, oral cleft defects, congenital heart defects, and controls. Adjusting for maternal race/ethnicity, education, study site, and folic acid supplementation, maternal nitrate intake of ≥ 5 mg per day from drinking water was associated with NTD-affected pregnancies [adjusted odds ratio (aOR) 1.43; 95% CI: 1.01, 2.04], although this association appeared to be specific to spina bifida (Table 2). Mothers of babies with spina bifida were 1.4 times more likely (95% CI: 0.86, 2.32) than control mothers to ingest between 0.91 and 4.9 mg nitrate per day and 2 times more likely (95% CI: 1.27, 3.22) to ingest ≥ 5 mg nitrate from drinking water around conception (*p* for trend = 0.003). During B1P3, mothers of babies with isolated limb deficiencies, cleft palate, and cleft lip without cleft palate were, respectively, 1.8 (95% CI: 1.05, 3.08), 1.9 (95% CI: 1.17, 3.09), and 1.8 times (95% CI: 1.08, 3.07) more likely than control mothers to ingest > 5.41 mg per day of nitrate from drinking water. We noted significant linear trends (*p* < 0.05) in the associations between maternal water nitrate and these defects in offspring (Table 2). In contrast, we saw minimal or no associations between maternal nitrate intake from drinking water and congenital heart defects in offspring. Restriction of analyses to women who reported drinking only tap water from municipal water supplies did not materially change the aORs associated with the highest tertile of water intake for spina bifida (aOR = 1.93; 95% CI: 0.99, 3.76), cleft lip without cleft palate (aOR = 1.96; 95% CI: 0.88, 4.36), or cleft palate (aOR = 1.55; 95% CI: 0.78, 3.10), but the aOR for any limb deficiency increased to 3.19 (95% CI: 1.09, 9.35) (see Supplemental Material, Table S1). A significant linear trend was observed for only cleft lip in relation to measured nitrate in drinking water among offspring of women who reported drinking municipal water (see Supplemental Material, Table S2). An aOR of 2.31 (95% CI: 1.20, 4.47) was noted for this defect among offspring of women who consumed water with nitrate levels > 15 mg/L relative to women who drank water with nitrate levels < 5 mg/L.

**Table 2:** Maternal daily nitrate intake from drinking water and selected birth defects in offspring.

Birth defect	Daily nitrate intake from water (mg/day)^*a*^	Cases [*n* (%)]	Controls [*n* (%)]	Unadjusted OR (95% CI)^*b*^	Adjusted OR (95%CI)^*b*^	*p*-Value for linear trend
Any NTD^*c*^	<0.91	67 (29.5)	367 (33.3)	1.00	1.00	0.038
	0.91–4.9	65 (28.6)	360 (32.7)	0.99 (0.68, 1.43)	1.00 (0.68, 1.45)
	≥5.0	95 (41.9)	374 (34.0)	1.39 (0.99, 1.96)	1.43 (1.01, 2.04)
Spina bifida^*c*^	<0.91	30 (22.4)	367 (33.3)	1.00	1.00	0.003
	0.91–4.9	42 (31.3)	360 (32.7)	1.43 (0.87, 2.33)	1.41 (0.86, 2.32)
	≥5.0	62 (46.3)	374 (34.0)	2.03 (1.28, 3.21)	2.02 (1.27, 3.22)
Anencephaly^*c*^	<0.91	31 (43.7)	367 (33.3)	1.00	1.00	0.348
	0.91–4.9	17 (23.9)	360 (32.7)	0.56 (0.30, 1.03)	0.58 (0.32, 1.08)
	≥5.0	23 (32.4)	374 (34.0)	0.73 (0.42, 1.27)	0.78 (0.44, 1.37)
Any limb deficiency^*d,e*^	<1.0	23 (24.5)	370 (33.5)	1.00	1.00	0.028
	1.0–5.41	29 (30.9)	367 (33.2)	1.27 (0.72, 2.24)	1.17 (0.66, 2.07)
	≥5.42	42 (44.7)	368 (33.3)	1.84 (1.08, 3.11)	1.79 (1.05, 3.08)
Any oral cleft defect^*e,f*^	<1.0	122 (29.4)	370 (33.5)	1.00	1.00	0.007
	1.0–5.41	120 (28.9)	366 (33.2)	0.99 (0.74, 1.33)	0.98 (0.73, 1.32)
	≥5.42	173 (41.7)	367 (33.3)	1.43 (1.09, 1.88)	1.45 (1.10, 1.92)
Cleft lip without cleft palate^*e,f*^	<1.0	24 (24.0)	370 (33.5)	1.00	1.00	0.019
	1.0–5.41	29 (29.0)	366 (33.2)	1.22 (0.70, 2.14)	1.13 (0.64, 1.99)
	≥5.42	47 (47.0)	367 (33.3)	1.97 (1.18, 3.30)	1.82 (1.08, 3.07)
Cleft palate^*e,f*^	<1.0	29 (25.2)	370 (33.5)	1.00	1.00	0.007
	1.0–5.41	32 (27.8)	366 (33.2)	1.12 (0.66, 1.88)	1.12 (0.66, 1.90)
	≥5.42	54 (47.0)	367 (33.3)	1.88 (1.17, 3.01)	1.90 (1.17, 3.09)
Conotruncal heart defects^*e,g*^	<1.0	58 (35.4)	370 (33.5)	1.00	1.00	0.403
	1.0–5.41	41 (25.0)	367 (33.2)	0.71 (0.47, 1.09)	0.72 (0.47, 1.11)
	≥5.42	65 (39.6)	368 (33.3)	1.13 (0.77, 1.65)	1.18 (0.80, 1.74)
Right ventricular outflow tract obstruction heart defects^*e,g*^	<1.0	36 (30.0)	370 (33.5)	1.00	1.00	0.083
	1.0–5.41	31 (25.8)	367 (33.2)	0.87 (0.53, 1.43)	0.89 (0.54, 1.48)
	≥5.42	53 (44.2)	368 (33.3)	1.48 (0.95, 2.32)	1.47 (0.93, 2.33)
Left ventricular outflow tract obstruction heart defects^*e,g*^	<1.0	44 (28.2)	370 (33.5)	1.00	1.00	0.522
	1.0–5.41	58 (37.2)	367 (33.2)	1.33 (0.88, 2.02)	1.31 (0.86, 2.00)
	≥5.42	54 (34.6)	368 (33.3)	1.23 (0.81, 1.88)	1.16 (0.75, 1.78)
Septal heart defects^*e,g*^	<1.0	203 (35.8)	370 (33.5)	1.00	1.00	0.853
	1.0–5.41	210 (37.0)	367 (33.2)	1.04 (0.82, 1.33)	0.92 (0.69, 1.22)
	≥5.42	154 (27.2)	368 (33.3)	0.76 (0.59, 0.98)	0.98 (0.71, 1.34)
^***a***^For NTDs, water nitrate intake 1 month preconception to 1 month postconception was estimated. For limb, oral cleft, and congenital heart defects, water nitrate intake 1 month preconception through the first trimester was estimated. ^***b***^Crude and adjusted ORs include only cases and controls with complete information for covariates. ^***c***^Adjusted for maternal race/ethnicity, education, study center, and folic acid supplementation. ^***d***^Adjusted for maternal race/ethnicity, education, age, multivitamin supplementation, and study center. ^***e***^Isolated defect. ^***f***^Adjusted for maternal race/ethnicity, education, age, folic acid supplementation, smoking, and study center. ^***g***^Adjusted for maternal race/ethnicity, education, multivitamin supplementation, smoking, and study center.						

No specific patterns of stronger associations between nitrosatable drug exposure (any versus none) and birth defects among women with higher daily intake of nitrate from drinking water were evident when aORs were stratified according to tertile of daily nitrate intake from drinking water (see Supplemental Material, Table S3). For several birth defect groups, the strongest associations with nitrosatable drug exposure were estimated for women in the lowest tertiles of estimated nitrate intake from drinking water [e.g., aORs = 2.54 (95% CI: 1.20, 5.37) and 2.89 (95% CI: 1.15, 7.25) for NTDs and cleft palate, respectively]. The CIs for the RERI and AP included 0, indicating no significant departures from additivity, and the *p*-values for the interaction terms for water and nitrosatable drug exposure were > 0.05, indicating no significant departures from multiplicative effects.

On the other hand, when estimated nitrate from drinking water and diet were combined with dietary nitrite intake to estimate total nitrite exposure from these sources, the strongest associations between nitrosatable drug exposure and several birth defects were observed among women with the highest estimated total nitrite exposure (the lower two tertiles of intake combined because of similarity of ORs) (see Supplemental Material, Table S4). Associations between nitrosatable drug exposure and birth defects were stronger in the highest tertile of total nitrite (vs. the lower two tertiles combined) for NTDs (aOR = 1.76; 95% CI: 0.90, 3.43 vs. aOR = 1.41; 95% CI: 0.87, 2.29), cleft lip without cleft palate (aOR = 2.01; 95% CI: 0.90, 4.48 vs. aOR = 0.80; 95% CI: 0.42, 1.52), cleft palate (aOR = 2.51; 95% CI: 1.24, 5.06 vs. aOR = 0.95; 95% CI: 0.55, 1.64), limb deficiencies (aOR = 1.64; 95% CI: 0.80, 3.35 vs. aOR = 1.00; 95% CI: 0.53, 1.89), atrioventricular septal defects (aOR = 5.10; 95% CI: 1.40, 18.6 vs. aOR = 1.93; 95% CI: 0.76, 4.87), and single ventricle (aOR = 3.25; 95% CI: 1.13, 9.31 vs. aOR = 0.74; 95% CI: 0.27, 2.02). Significant departures from additivity were noted for the joint estimated effects of total nitrite intake and nitrosatable drug exposures for cleft lip, cleft palate, limb deficiencies, and single ventricle; multiplicative interaction was also present in this association with cleft palate (see Supplemental Material, Table S4).

## Discussion

Results from this large population-based case–control study suggest that prenatal nitrate intake from drinking water is associated with NTDs, oral cleft defects, and limb deficiencies in offspring. Previous publications that have reported significant associations between drinking-water nitrates and birth defects hypothesized that nitrate might act as a teratogen through its contribution to the endogenous formation of *N*-nitroso compounds (Croen et al. 2001; Dorsch et al. 1984). In the present study, however, higher daily intake of nitrate from drinking water did not strengthen associations between nitrosatable drugs and the various birth defects examined. On the other hand, associations between nitrosatable drugs and birth defects were stronger among women in the highest tertile of estimated total nitrite intake, a measure based on intake of dietary nitrite and nitrate from diet and drinking water. In this study, nitrate levels in the drinking water tended to be low, with a median contribution of nitrate per day from this source of 6% in the study population. In a recent review, the World Health Organization (2011) noted that the contribution of drinking water to nitrate intake is usually < 14%.

Previous studies have assigned exposure based on measured nitrate in drinking water instead of estimating daily ingestion. For women who drank water from groundwater sources, measured levels of total nitrate as low as 5–15 mg/L have been significantly associated with birth defects (Dorsch et al. 1984) including anencephaly (Croen et al. 2001). Although we noted significant ORs in the relation between measured nitrate levels at ≥ 5 mg/L and several birth defects, we saw a significant linear trend only for cleft lip without cleft palate in our study population. Other studies have reported elevated, but not statistically significant, ORs for central nervous system defects (Arbuckle et al. 1988) and NTDs (Brender et al. 2004) for measured nitrate levels respectively at 26 mg/L (relative to 0.1 mg/L) and ≥ 3.5 mg/L (relative to < 3.5 mg/L). Positive associations were restricted to groundwater drinkers in several of these studies, and the authors suggested that other agents correlated with nitrate in groundwater might be responsible for the associations noted (Croen et al. 2001; Dorsch et al. 1984).

In contrast to findings from a study of nitrosatable drugs and NTDs in Mexican Americans (Brender et al. 2004), in the present study, higher intake of nitrate from drinking water did not strengthen the association between nitrosatable drug use and NTDs, nor was this pattern noted for the other birth defects examined. In two earlier studies (Brender et al. 2011b, 2012) of NBDPS, which included participants from all 10 sites, associations between prenatal nitrosatable drug exposure and several birth defects, including NTDs, cleft palate, conotruncal heart defects, atrioventricular septal defects, and single ventricle defects were stronger among women with the highest estimated intake of nitrite from dietary sources than in women with lower estimated dietary intakes. Similarly in the present study, associations between nitrosatable drug use and several of the same defects were stronger with higher estimated total nitrite intake, which included intake from drinking-water as well as dietary sources. Water nitrate contributed, on average (median), approximately 3% of total daily nitrite in the present study population. Therefore, water nitrate might be associated with birth defects for reasons other than its contribution to the endogenous formation of *N*-nitroso compounds. Nitrate has been found to occur with other contaminants in drinking water, especially in conjunction with pesticides, arsenic and other trace metals, and water disinfection by-products (Toccalino et al. 2012).

In the present study, we focused on nitrate contamination in drinking-water sources without examining the presence of other water contaminants. Another study limitation was the potential for measurement errors in nitrate content of drinking-water sources and daily consumption of water nitrate. Estimates of nitrate in sources from public water systems were based on data from routine monitoring in which we linked addresses to the most time-relevant sample results available. Our approach for assigning nitrate levels to municipal drinking-water sources was not validated, although we developed and followed a detailed set of standard operating procedures for such assignment (Griesenbeck et al. 2009a). The high percentage of bottled water users presented a challenge in exposure assessment because participants were not specifically questioned about types of bottled water consumed. Therefore, nitrate content from this source was estimated from nitrate measured in bottled water samples from neighborhood grocery store surveys. However, associations noted between nitrate intake from drinking-water nitrate and birth defects changed very little when the analysis was restricted to women who reported drinking tap water from municipal water supplies only. We estimated nitrate content in private wells through complex models that took into account local conditions; however, this modeling effort was restricted to private well users in Texas. Although it is possible that some participants might have not accurately recalled the types and amounts of water that they consumed during early pregnancy, Shimokura et al. (1998) found good agreement (Pearson’s *r* = 0.78) between a questionnaire on past use and a 3-day water diary for drinking-water intake in a sample of pregnant women. Given that all exposure assessments in this study of drinking-water nitrate were completed with the study teams blinded to case–control status, misclassification of daily nitrate intake from drinking water would most likely be nondifferential and have led to an underestimation of the true ORs. Measurement error might have also occurred with the estimation of dietary intake of nitrate and nitrite, and this limitation is discussed in detail in previous publications (Brender et al. 2011a, 2012) along with the potential for bias in participant recall of drugs taken during early pregnancy.

## Conclusion

In this large, population-based case–control study, women who had babies with NTDs, limb deficiencies, and oral cleft defects were significantly more likely than control mothers to ingest ≥ 5 mg of nitrate per day from drinking water. However, study findings suggest that endogenous formation of *N*-nitroso compounds might not be the underlying mechanism for potential teratogenesis with this water contaminant, because higher intake of nitrate from drinking water did not strengthen associations between prenatal nitrosatable drug exposure and birth defects in offspring. Given that nitrate contamination occurs in conjunction with other water contaminants, future studies of birth defects might focus on prenatal exposure to mixtures of contaminants in drinking water.

## Supplemental Material

(360 KB) PDF
