# Supplementary material for: Prenatal Nitrate Intake from Drinking Water and Selected Birth Defects in Offspring of Participants in the National Birth Defects Prevention Study
Source: Environ Health Perspect. 2013 Jun 14;121(9):1083–9. doi: 10.1289/ehp.1206249 (PMC3764078; doi:10.1289/ehp.1206249)
Supplement: (360 KB) PDF [file ehp.1206249.s001.pdf]

Supplemental Material

Prenatal Nitrate Intake from Drinking Water and Selected Birth Defects in Offspring of Participants in the National Birth Defects Prevention Study

Jean D. Brender, Peter J. Weyer, Paul A. Romitti, Binayak P. Mohanty, Mayura U. Shinde, Ann M. Vuong, Joseph R. Sharkey, Dipankar Dwivedi, Scott A. Horel, Jiji Kantamneni, John C. Huber Jr., Qi Zheng, Martha M. Werler, Katherine E. Kelley, John S. Griesenbeck, F. Benjamin Zhan, Peter H. Langlois, Lucina Suarez, Mark A. Canfield, and the National Birth Defects Prevention Study

Table of Contents

	Page
Supplemental Material, Table S1. Maternal daily nitrate intake from drinking water and selected birth defects in offspring among participants who drank only tap water from municipal water supplies	2
Supplemental Material, Table S2. Nitrate in municipal drinking water source of tap water drinkers and selected birth defects in offspring, Iowa and Texas participants of the National Birth Defects Prevention Study	4
Supplemental Material, Table S3. Associations between nitrosatable drug use (versus no use) and birth defects according to estimated nitrate intake from drinking water during early pregnancy	6
Supplemental Material, Table S4. Associations between nitrosatable drug use (versus no use) and birth defects according to estimated total nitrite intake from diet and drinking water during early pregnancy	8

Supplemental Material, Table S1. Maternal daily nitrate intake from drinking water and selected birth defects in offspring among participants who only drank tap water from municipal water supplies

Type of Birth defect	Daily nitrate intake from water[a] (mg/day)	Cases No. (%)	Controls No. (%)	Unadjusted OR[b] (95%CI)	Adjusted OR[b] (95%CI)	*p*-value for linear trend
Any neural tube defect[c]						
	< 0.91	25 (16.7)	125 (18.9)	1.00	1.00	
	0.91 – 4.9	38 (25.3)	199 (30.2)	0.95 (0.55, 1.66)	0.91 (0.52, 1.60)	
	≥ 5.0	87 (58.0)	336 (50.9)	1.29 (0.79, 2.11)	1.25 (0.76, 2.06)	0.230
Spina bifida[c]	< 0.91	12 (12.1)	125 (18.9)	1.00	1.00	
	0.91 – 4.9	27 (27.3)	199 (30.2)	1.41 (0.69, 2.89)	1.40 (0.68, 2.89)	
	≥ 5.0	60 (60.6)	336 (50.9)	1.86 (0.97, 3.57)	1.93 (0.99, 3.76)	0.037
Anencephaly[c]	< 0.91	9 (24.3)	125 (18.9)	1.00	1.00	
	0.91 – 4.9	9 (24.3)	199 (30.2)	0.63 (0.24, 1.63)	0.59 (0.23, 1.56)	
	≥ 5.0	19 (51.4)	336 (50.9)	0.79 (0.35, 1.78)	0.69 (0.30, 1.61)	0.510
Any limb deficiency[d,e]	< 1.0	4 (6.6)	114 (17.2)	1.00	1.00	
	1.0 – 5.41	19 (31.2)	215 (32.4)	2.52 (0.84, 7.58)	2.43 (0.80, 7.43)	
	≥ 5.42	38 (62.3)	335 (50.5)	3.23 (1.13, 9.26)	3.19 (1.09, 9.35)	0.030
Any oral cleft defect[e,f]	< 1.0	47 (16.9)	114 (17.3)	1.00	1.00	
	1.0 – 5.41	74 (26.5)	214 (32.4)	0.84 (0.55, 1.29)	0.80 (0.52, 1.25)	
	≥ 5.42	158 (56.6)	333 (50.4)	1.15 (0.78, 1.70)	1.15 (0.77, 1.72)	0.191
Cleft lip without cleft palate[e,f]	< 1.0	8 (11.6)	114 (17.3)	1.00	1.00	
	1.0 – 5.41	15 (21.7)	214 (32.4)	1.00 (0.41, 2.43)	0.96 (0.39, 2.37)	
	≥ 5.42	46 (66.7)	333 (50.4)	1.97 (0.90, 4.30)	1.96 (0.88, 4.36)	0.022
Cleft palate[e,f]	< 1.0	12 (15.0)	114 (17.3)	1.00	1.00	
	1.0 – 5.41	20 (25.0)	214 (32.4)	0.89 (0.42, 1.88)	0.93 (0.43, 2.02)	
	≥ 5.42	48 (60.0)	333 (50.4)	1.37 (0.70, 2.67)	1.55 (0.78, 3.10)	0.092
Conotruncal[e,g]	< 1.0	15 (15.8)	114 (17.2)	1.00	1.00	
	1.0 – 5.41	25 (26.3)	215 (32.4)	0.88 (0.45, 1.74)	0.92 (0.46, 1.83)	
	≥ 5.42	55 (57.9)	335 (50.5)	1.25 (0.68, 2.29)	1.29 (0.69, 2.41)	0.252

Type of Birth defect	Daily nitrate intake from water[a] (mg/day)	Cases No. (%)	Controls No. (%)	Unadjusted OR[b] (95%CI)	Adjusted OR[b] (95%CI)	*p*-value for linear trend
Right ventricular outflow tract obstruction[e,g]	< 1.0	9 (11.1)	114 (17.2)	1.00	1.00	
	1.0 – 5.41	24 (29.6)	215 (32.4)	1.41 (0.64, 3.14)	1.46 (0.65, 3.28)	
	≥ 5.42	48 (59.3)	335 (50.5)	1.81 (0.86, 3.82)	1.90 (0.89, 4.01)	0.076
Left ventricular outflow tract obstruction[e,g]	< 1.0	17 (17.4)	114 (17.2)	1.00	1.00	
	1.0 – 5.42	33 (33.7)	215 (32.4)	1.03 (0.55, 1.93)	1.12 (0.60, 2.12)	
	> 5.42	48 (49.0)	335 (50.5)	0.96 (0.53, 1.74)	1.06 (0.58, 1.92)	0.944
Septal defects[e,g]	< 1.0	55 (17.8)	114 (17.2)	1.00	1.00	
	1.0 – 5.42	117 (37.9)	215 (32.4)	1.13 (0.76, 1.67)	1.09 (0.72, 1.63)	
	> 5.42	137 (44.3)	335 (50.5)	0.85 (0.58, 1.24)	0.87 (0.59, 1.30)	0.311

[a] For neural tube defects, water nitrate intake one month preconception to one month post-conception was estimated. For limb, oral cleft and congenital heart defects, water nitrate intake during one month preconception through the first trimester was estimated.

[b] Crude and adjusted odds ratio include only cases and controls with complete information for covariates.

[c] Adjusted for maternal race/ethnicity, education, study center and folic acid supplementation.

[d] Adjusted for maternal race/ethnicity, education, age, multivitamin supplementation, and study center.

[e] Isolated defect

[f] Adjusted for maternal race/ethnicity, education, age, folic acid supplementation during the first trimester, maternal smoking, and study center.

[g] Adjusted for maternal race/ethnicity, education, multivitamin use during the first trimester, maternal smoking, and study center.

Supplemental Material, Table S2. Nitrate in municipal drinking water source of tap water drinkers[a] and selected birth defects in offspring, Iowa and Texas participants of the National Birth Defects Prevention Study

Type of birth defect	Nitrate[b] (mg/L)	Cases No. (%)	Controls No. (%)	Unadjusted OR[c] (95% CI)	Adjusted OR[c] (95% CI)	*p*-value for linear trend
Any neural tube defect[d]	< 5.0	67 (43.2)	353 (51.6)	1.00	1.00	
	5.0 –15.0	72 (46.5)	205 (30.0)	1.85 (1.27, 2.69)	1.82 (1.24, 2.67)	
	> 15.0	16 (10.3)	126 (18.4)	0.67 (0.37, 1.20)	0.66 (0.36, 1.21)	
						0.794
Spina bifida[d]	< 5.0	40 (38.8)	353 (51.6)	1.00	1.00	
	5.0 –15.0	52 (50.5)	205 (30.0)	2.24 (1.43, 3.50)	2.42 (1.52, 3.84)	
	> 15.0	11 (10.7)	126 (18.4)	0.77 (0.38, 1.55)	0.69 (0.33, 1.42)	
						0.501
Anencephaly[d]	< 5.0	20 (52.6)	353 (51.6)	1.00	1.00	
	5.0 –15.0	13 (34.2)	205 (30.0)	1.12 (0.55, 2.30)	0.92 (0.44, 1.93)	
	> 15.0	5 (13.2)	126 (18.4)	0.70 (0.26, 1.91)	0.85 (0.29, 2.49)	
						0.736
Any limb deficiency[e,f]	< 5.0	28 (44.4)	337 (48.8)	1.00	1.00	
	5.0 –15.0	23 (36.5)	229 (33.2)	1.21 (0.68, 2.15)	1.08 (0.59, 1.99)	
	> 15.0	12 (19.1)	124 (18.0)	1.16 (0.57, 2.36)	1.53 (0.71, 3.33)	
						0.329
Any oral cleft defect[f,g]	< 5.0	127 (44.9)	335 (48.8)	1.00	1.00	
	5.0 –15.0	109 (38.5)	229 (33.3)	1.26 (0.92, 1.71)	1.22 (0.89, 1.68)	
	> 15.0	47 (16.6)	123 (17.9)	1.01 (0.68, 1.49)	1.18 (0.78, 1.79)	
						0.269
Cleft lip without cleft palate[f,g]	< 5.0	29 (40.3)	335 (48.8)	1.00	1.00	
	5.0 –15.0	22 (30.6)	229 (33.3)	1.11 (0.62, 1.98)	1.07 (0.59, 1.96)	
	> 15.0	21 (29.2)	123 (17.9)	1.97 (1.08, 3.59)	2.31 (1.20, 4.47)	
						0.026
Cleft palate[f,g]	< 5.0	32 (40.0)	335 (48.8)	1.00	1.00	
	5.0 –15.0	36 (45.0)	229 (33.3)	1.65 (0.99, 2.73)	1.75 (1.03, 2.98)	
	> 15.0	12 (15.0)	123 (17.9)	1.02 (0.51, 2.05)	1.10 (0.52, 2.29)	
						0.344

Type of birth defect	Nitrate[b] (mg/L)	Cases No. (%)	Controls No. (%)	Unadjusted OR[c] (95% CI)	Adjusted OR[c] (95% CI)	p-value for linear trend
Conotruncal heart defects[f,h]	< 5.0	50 (49.0)	337 (48.8)	1.00	1.00	
	5.0 –15.0	39 (38.2)	229 (33.2)	1.15 (0.73, 1.80)	1.15 (0.72, 1.83)	
	> 15.0	13 (12.8)	124 (18.0)	0.71 (0.37, 1.35)	0.70 (0.35, 1.37)	
						0.536
Right ventricular outflow tract obstruction[f,h]	< 5.0	33 (39.3)	337 (48.8)	1.00	1.00	
	5.0 –15.0	32 (38.1)	229 (33.2)	1.43 (0.85, 2.39)	1.44 (0.83, 2.51)	
	> 15.0	19 (22.6)	124 (18.0)	1.56 (0.86, 2.85)	1.41 (0.74, 2.70)	
						0.210
Left ventricular outflow tract obstruction[f,h]	< 5.0	44 (44.4)	337 (48.8)	1.00	1.00	
	5.0 –15.0	30 (30.3)	229 (33.2)	1.00 (0.61, 1.64)	1.12 (0.67, 1.86)	
	> 15.0	25 (25.3)	124 (18.0)	1.54 (0.91, 2.63)	1.37 (0.78, 2.39)	
						0.278
Septal defects[f,h]	< 5.0	201 (62.8)	337 (48.8)	1.00	1.00	
	5.0 –15.0	79 (24.7)	229 (33.2)	0.58 (0.42, 0.79)	0.58 (0.39, 0.88)	
	> 15.0	40 (12.5)	124 (18.0)	0.54 (0.36, 0.80)	0.81 (0.47, 1.40)	
						0.103

OR (odds ratio); CI (confidence interval). [a] Women who reported drinking any municipal tap water during one month preconception to one month post-conception for neural tube defects, and one month preconception through the first trimester for limb, oral cleft and congenital heart defects. [b] For neural tube defects, nitrate in drinking water one month preconception to one month post-conception was estimated. For limb, oral cleft and congenital heart defects, nitrate in drinking water during one month preconception through the first trimester was estimated. [c] Crude and adjusted odds ratio include only cases and controls with complete information for covariates. [d] Adjusted for maternal race/ethnicity, education, study center and folic acid supplementation during period one month before through one month post-conception. [e] Adjusted for maternal race/ethnicity, education, age, multivitamin use during the first trimester, and study center. [f] Isolated defect. [g] Adjusted for maternal race/ethnicity, education, age, folic acid supplementation during the first trimester, maternal smoking, and study center. [h] Adjusted for maternal race/ethnicity, education, multivitamin use during the first trimester, maternal smoking, and study center.

Supplemental Material, Table S3. Associations between nitrosatable drug use (versus no use) and birth defects according to estimated nitrate intake from drinking water during early pregnancy

		Nitrosatable drug exposure during the first trimester							
		Cases		Controls					
Congenital malformation	Nitrate[a] (mg/day)	Yes	No	Yes	No	Unadjusted OR[b] (95% CI)	Adjusted OR[b] (95% CI)	Attributable proportion due to interaction	p-value for multiplicative interaction term
Any neural tube defect[c]	< 0.91	13	54	37	330	2.15 (1.07, 4.30)	2.54 (1.20, 5.37)		
	0.91 – 4.99	11	54	59	301	1.04 (0.51, 2.11)	1.26 (0.60, 2.65)		
	≥ 5.0	20	75	61	313	1.37 (0.78, 2.41)	1.20 (0.66, 2.19)		
								-0.29 (-1.27, 0.69)	0.283
Any limb deficiency[d,e]	< 1.0	3	19	66	297	0.71 (0.20, 2.47)	0.72 (0.19, 2.70)		
	1.0 – 5.41	8	20	79	280	1.42 (0.60, 3.34)	1.12 (0.45, 2.79)		
	≥ 5.42	12	29	86	276	1.33 (0.65, 2.71)	1.20 (0.57, 2.53)		
								0.32 (-0.40, 1.03)	0.440
Cleft lip with or without cleft palate[e,f]	< 1.0	16	75	66	297	0.96 (0.53, 1.75)	0.97 (0.52, 1.84)		
	1.0 – 5.41	20	66	78	280	1.09 (0.62, 1.90)	0.99 (0.54, 1.80)		
	≥ 5.42	28	88	85	276	1.03 (0.63, 1.69)	1.01 (0.60, 1.69)		
								-0.003 (-0.69, 0.68)	0.988
Cleft palate [e,f]	< 1.0	10	18	66	297	2.50 (1.10, 5.66)	2.89 (1.15, 7.25)		
	1.0 – 5.41	5	26	78	280	0.69 (0.26, 1.86)	0.60 (0.20, 1.78)		
	≥ 5.42	16	38	85	276	1.37 (0.73, 2.57)	1.31 (0.67, 2.54)		
								-0.24 (-1.17, 0.69)	0.391
Conotruncal heart defect[e,g]	< 1.0	12	45	66	297	1.20 (0.60, 2.39)	1.39 (0.66, 2.91)		
	1.0 – 5.41	7	32	79	280	0.78 (0.33, 1.82)	0.86 (0.35, 2.13)		
	≥ 5.42	10	52	86	276	0.62 (0.30, 1.27)	0.59 (0.28, 1.23)		
								-0.88 (-2.56, 0.80)	0.182
Right ventricular outflow tract obstruction[e,g]	< 1.0	8	27	66	297	1.33 (0.58, 3.07)	1.36 (0.56, 3.31)		
	1.0 – 5.41	10	21	79	280	1.69 (0.76, 3.73)	2.25 (0.94, 5.40)		
	≥ 5.42	8	45	86	276	0.57 (0.26, 1.26)	0.56 (0.25, 1.28)		
								-1.24 (-3.32, 0.84)	0.095
Left ventricular outflow tract obstruction[e,g]	< 1.0	9	35	66	297	1.16 (0.53, 2.52)	1.06 (0.47, 2.39)		
	1.0 – 5.41	11	45	79	280	0.87 (0.43, 1.75)	0.81 (0.39, 1.69)		
	≥ 5.42	12	39	86	276	0.99 (0.49, 1.97)	0.83 (0.41, 1.70)		
								-0.15 (-1.29, 0.99)	0.892

Congenital malformation	Nitrate[a] (mg/day)	Nitrosatable drug exposure during the first trimester Cases Yes	No	Controls Yes	No	Unadjusted OR[b] (95% CI)	Adjusted OR[b] (95% CI)	Attributable proportion due to interaction	*p*-value for multiplicative interaction term
Septal heart defect[e,g]	< 1.0	47	154	66	297	1.37 (0.90, 2.09)	1.53 (0.97, 2.42)		
	1.0 – 5.41	43	157	79	280	0.97 (0.64, 1.48)	1.03 (0.66, 1.61)		
	≥ 5.42	41	110	86	276	1.20 (0.78, 1.84)	1.19 (0.76, 1.88)		
								-0.20 (-0.93, 0.53)	0.655

OR (odds ratio); CI (confidence interval).

[a] For neural tube defects, water nitrate intake one month preconception to one month post-conception was estimated. For limb, oral cleft and congenital heart defects, water nitrate intake one month preconception through the first trimester was estimated.

[b] Crude and adjusted odds ratio (OR) include only cases and controls with complete information for covariates.

[c] Adjusted for maternal race/ethnicity, education, study center and folic acid supplementation.

[d] Adjusted for maternal race/ethnicity, education, age, multivitamin supplementation, and study center.

[e] Isolated defect.

[f] Adjusted for maternal race/ethnicity, education, age, folic acid supplementation, smoking, and study center.

[g] Adjusted for maternal race/ethnicity, education, multivitamin supplementation, smoking, and study center.

Supplemental Material, Table S4. Associations between nitrosatable drug use (versus no use) and birth defects according to estimated total nitrite intake from diet and drinking water during early pregnancy

Type of birth defect	Total nitrite intake[a] (mg/day)	Nitrosatable drug exposure during the first trimester: Cases Yes	Cases No	Controls Yes	Controls No	Unadjusted OR[b] (95% CI)	Adjusted OR[b] (95% CI)	Attributable proportion due to interaction[c]	*p*-value for multiplicative interaction term
Any neural tube defect[d]	≤ 4.74	27	112	111	597	1.30 (0.81, 2.07)	1.41 (0.87, 2.29)		
	> 4.74	16	67	41	313	1.82 (0.97, 3.44)	1.76 (0.90, 3.43)		
								0.31 (-0.21, 0.83)	0.396
Cleft lip without cleft palate[e]	≤ 4.78	14	52	159	536	0.91 (0.49, 1.68)	0.80 (0.42, 1.52)		
	> 4.78	13	23	65	284	2.47 (1.19, 5.13)	2.01 (0.90, 4.48)		
								0.60 (0.15, 1.05)	0.074
Cleft lip with cleft palate[e]	≤ 4.78	29	134	159	536	0.73 (0.47, 1.13)	0.76 (0.48, 1.21)		
	> 4.78	12	52	65	284	1.01 (0.51, 2.00)	1.13 (0.54, 2.37)		
								0.34 (-0.41, 1.09)	0.533
Cleft palate[e]	≤ 4.78	20	69	159	536	0.98 (0.58, 1.66)	0.95 (0.55, 1.64)		
	> 4.78	19	30	65	284	2.77 (1.47, 5.22)	2.51 (1.24, 5.06)		
								0.63 (0.32, 0.95)[h]	0.019
Any limb deficiency[f]	≤ 4.78	14	52	161	537	0.90 (0.49, 1.66)	1.00 (0.53, 1.89)		
	> 4.78	14	35	65	283	1.74 (0.89, 3.42)	1.64 (0.80, 3.35)		
								0.47 (0.05, 0.89)	0.154
Conotruncal heart defect[g]	≤ 4.78	33	114	161	537	0.97 (0.63, 1.48)	0.98 (0.64, 1.52)		
	> 4.78	13	64	65	283	0.88 (0.46, 1.70)	0.80 (0.40, 1.61)		
								-0.13 (-1.02, 0.76)	0.769
Right ventricular outflow tract obstruction[g]	≤ 4.78	31	88	161	537	1.17 (0.75, 1.83)	1.15 (0.72, 1.82)		
	> 4.78	11	54	65	283	0.89 (0.44, 1.79)	0.83 (0.40, 1.74)		
								-0.26 (-1.26, 0.74)	0.565
Left ventricular outflow tract obstruction[g]	≤ 4.78	28	109	161	537	0.86 (0.55, 1.35)	0.82 (0.52, 1.30)		
	> 4.78	14	56	65	283	1.09 (0.57, 2.07)	0.96 (0.49, 1.92)		
								0.19 (-0.47, 0.85)	0.578
Septal heart defect[g]	≤ 4.78	125	373	161	537	1.12 (0.85, 1.46)	1.19 (0.90, 1.58)		

Type of birth defect	Total nitrite intake[a] (mg/day)	Nitrosatable drug exposure during the first trimester: Cases Yes	Cases No	Controls Yes	Controls No	Unadjusted OR[b] (95% CI)	Adjusted OR[b] (95% CI)	Attributable proportion due to interaction[c]	*p*-value for multiplicative interaction term
	> 4.78	63	244	65	283	1.12 (0.76, 1.65)	1.09 (0.72, 1.64)		
								0.03 (-0.42, 0.47)	0.955
Atrioventricular septal defect[g]	≤ 4.78	8	13	161	537	2.05 (0.84, 5.04)	1.93 (0.76, 4.87)		
	> 4.78	7	6	65	283	5.08 (1.65, 15.6)	5.10 (1.40, 18.6)		
								0.50 (-0.17, 1.16)	0.230
Single ventricle[g]	≤ 4.78	5	24	161	537	0.69 (0.26, 1.85)	0.74 (0.27, 2.02)		
	> 4.78	7	11	65	283	2.77 (1.03, 7.42)	3.25 (1.13, 9.31)		
								0.75 (0.32, 1.18)	0.063

OR (odds ratio); CI (confidence interval).

[a]Total nitrite intake = dietary nitrite intake + 5% (dietary nitrate intake + nitrate from drinking water). Contribution from nitrate in drinking water estimated for one month prior through one month postconception for NTDs and one month prior to conception through the first trimester for the other congenital malformations. [b]Crude and adjusted odds ratio include only cases and controls with complete information for covariates and calorie intake between 500-5000 kcal. [c]Interaction assessed on dichotomized categories of total nitrite intake: highest tertile versus the two lower tertiles of intake combined. [d]Adjusted for maternal race/ethnicity, education, study center, folic acid supplementation during period one month before through one month post conception, and caloric intake. [e]Adjusted for maternal race/ethnicity, education, age, folic acid supplementation during the first trimester, maternal smoking, study center, and caloric intake. [f]Adjusted for maternal race/ethnicity, education, age, multivitamin use during the first trimester, study center, and caloric intake. [g]Adjusted for maternal race/ethnicity, education, multivitamin use during the first trimester, maternal smoking, study center, and caloric intake. [h]Significant relative excess risk due to interaction (RERI) 1.60 (0.11, 3.09) was observed for cleft palate.
